# Navigating uncertainty and negotiating trust in judicial deliberations

**DOI:** 10.3389/fsoc.2024.1423885

**Published:** 2024-11-12

**Authors:** Stina Bergman Blix, Nina Törnqvist

**Affiliations:** Department of Sociology, Uppsala University, Uppsala, Sweden

**Keywords:** judicial deliberation, trust, epistemic emotions, judges, legal decision making, social interaction, legal independence, uncertainty

## Abstract

Autonomy and independence are key features of legal decision-making. Yet, decision-making in court is fundamentally interactional and collective, both during the information gathering phase of hearings, and in evaluations during deliberations. Depending on legal system and type of court, deliberations can include different constellations of lay judges, jurors, or judge panels. In this article, we explore the collective dynamic of knowledge acquisition in legal decision-making, by analysing their emotional undercurrents. We show how judges balance uncertainty and certainty in legal deliberation, elaborating on (1) trust; (2) uncertainty exchange, and; (3) certainty as an agile emotion. Theoretically, the article combines an emotive-cognitive judicial framework, which understands emotion and reason as intersecting and continuous, with social interactionist theory. The analysis builds on extensive ethnographic fieldwork in Sweden, including shadowing and interviews with judges as well as observations during court proceedings and deliberations. The article actualizes the joint accomplishment of legal independence, and contributes with a nuanced account of how the decision-making process unfolds in legal deliberations.

## Introduction

The image of the judge is singular, the lone arbiter in her chambers or on the bench taking independent decisions. The stand-alone image of the judge rhymes with a founding principle of a democratic rule of law, both in terms of the judicial power as independent from the legislative and executive powers, and as a professional core, the judge must ‘have the courage to arrive at uncomfortable decisions and be able to withstand pressure of public opinion’ (Hirschfeldt, 2011: 7). Judicial independence tends to emphasize professional autonomy for the court corps rather than for individual judges (Ställvik, 2009). This focus on judges as a corps can be linked to the *image* of the judge as a mechanical and rational applier of legal rules, making individual autonomy a non-issue. Previous studies of legal *practice*, have found judges to emphasize the craftwork of judging, conveying that empathy, impartiality and irreproachability has more bearing on their work than legal expertise (Ställvik, 2009; Tata, 2007). Consequently, legal decision-making becomes associated with craftwork and empathic attuning rather than calculation and computation (Hutton, 2006; Ställvik, 2009; Tata, 2007; van Oorschot, 2021), paralleled by an upsurge of studies on the emotional dimensions of judicial work (Bergman Blix and Wettergren, 2018; Roach Anleu and Mack, 2021; Bandes et al., 2021). The judge as a craftsperson humanizes judges’ work, but still emphasizes the individual rather than the collective. At the same time, knowledge processes are fundamentally social. What constitutes knowledge, how to evaluate knowledge, and make decisions are formed in interaction between people (Blumer, 1969; Durkheim, 2008). This is particularly evident in courts, where decision-making is inherently interactional and collective, both during the information gathering phase of hearings, and in evaluations during deliberations (Bergman Blix, 2022b). In this article, our aim is to explore the collective dynamic of knowledge acquisition in legal decision-making, by analysing their emotional undercurrents. We show how judges balance uncertainty and certainty in legal deliberation, focusing (1) trust; (2) uncertainty exchange, referring to judges sharing and discussing legal or factual issues that they feel uncertain about, and; (3) certainty as an agile emotion. Despite high demands on independence, trust or mistrust form the basis for collective deliberations in court developing into group solidarity or alienation.

In the Swedish court system, the professional judge rules in a panel with three lay judges in the lower court, while the appellate court panel consists of three professional judges and two lay judges. The professional judge presides in trials and the panel decides on both guilt and sanction in the same trial. Deliberations are confidential and usually take place in the court room when the parties have left directly after a hearing. In larger trials going on for several days or weeks, the panel can start to deliberate after each day, but then meet at a separate occasion after the full trial is over. The decision is reached by a majority vote and potential disagreements are incorporated as dissenting opinions at the end of the written verdict.^1^ The legal verdict can sometimes be delivered orally, but is always delivered in written form including motivations. In the district court, the professional judge writes the judgment, and in the appellate court, the reporting judge is responsible for writing the draft that is then circulated between the three professional judges.

### How judges decide – previous research on legal decision-making

How do judges think and decide on cases? This engaging question echoes throughout the ample reservoir of socio-legal studies interested in understanding how legal decisions come about. In his seminal work, *How judges think*, American judge Posner (2008: 111) suggests that judges, like all human beings, are prone to ‘rationalization’. This idea constitutes a hallmark of the positivistic legal tradition, in which legal decision-making is portrayed as a rational, rather mechanistic enterprise of information gathering and interpretation. In this framework, legal decisions are *reasoned* decisions purely based on the facts of the case and the law. However, this paradigm has been challenged based on findings in psychology, neuroscience and philosophy focusing on the role emotions play in rational inferences.

A wealth of psychological studies has showed that emotions have pervasive impact on all kinds of decision-making processes (Lerner et al., 2015). Besides elucidating emotions as drivers of decision-making, these studies also demonstrate that emotions affect the content of thought, the depth of thought and the content of implicit goals. Focusing on judges, Bennett and Broe (2010) argue that emotions facilitate legal decision-making as emotions enable learning and memorising, which is essential for acquiring experience. In addition, they maintain that emotions are of particular importance for hunches, gut feelings and feeling that a decision is right. Informed by experimental psychological research, legal scholars have argued that judges tend to intuitive reasoning when they make decisions and that judges, like all human beings, resort to mental shortcuts, involving anchoring, statistical inferences, hindsight and confirmation biases (Guthrie et al., 2007; Rachlinski and Wistrich, 2017). This aligns with research arguing that most legal decisions are part of a routine professional practice that demand little reflection where judges tend to ‘know’ the right decision before they have assessed the case properly. Education and working experience generate a decision-making process that is mainly intuitive and unarticulated.

Socio-legal research demonstrates how the nexus of legal principles (impartiality, objectivity, neutrality, independence, detachment and dispassion) form a normative framework that shapes legal decision-making. Through empirical work on Australian judges, Roach Anleu and Mack (2021) show how the legal concept of impartiality mobilize ambitions among judges to keep an open mind and an ability to put legally irrelevant information, attitudes or emotions aside. In a similar vein, Sutton (2010: 875) highlights open-mindedness, humility and the intent to get it right as central aspects of legal temperament. In a recent article on judicial independence, Jamieson (2021) argues that while this principle relies on collaboration with other legal actors, for the individual judge, it serves as a commitment to carry out their duties in particular ways. Realized in legal practice, independence becomes an act of balancing rather than separating rationality, intuition and emotions (Jamieson, 2021: 146). Pointing toward the emotional aspect of these professional norms, Bergman Blix and Minissale (2022) highlight that judges and other legal actors need to *feel* committed to these principles for them to be actualised.

Research focusing on social and collective backdrops of legal decision-making have to a large extent looked at outcomes in terms of sentencing rather than the collective process itself, by pinpointing the importance of local legal cultures (Eisenstein and Jacob, 1977) and legal training and legal socialization (Steffensmeier and Hebert, 1999: 1187) to understand legal decision-making practices and processes. Collegiality has been shown to play down personal and political orientations (Edwards, 2003), and safeguard against judge’s individual preferences (Sutton, 2010), but collective aspects, or ‘panel effects’, such as voting order also indicate strong norms of consensus among the judges (Fischman, 2013).

To sum up, although previous research brings forward emotional as well as collective dimensions of legal decision-making, micro sociological studies that combine the two, exploring how judges emotionally and collectively arrive at a decision are scarce (Edwards, 2003). Analysing empirical data from actual deliberations in Swedish district and appellate courts provide a unique opportunity to advance our knowledge on how judges’ decisions form and inform epistemic and collective emotions in exchange with their close peers.

### Epistemic emotions and social interaction

Our theoretical vantage point assumes that rational action at large demands emotional support and motivation. As a state of mind, rationality, involves feelings, such as ease and confidence (James, 1879; Barbalet, 2011). These feelings are associated with an absence of obstacles implying that a rational decision is perceived as effortless or evident.^2^ Rationality is thus not emotionless, but demands smooth emotions, emotions that are not perceived as disruptive or ‘emotional’, but instead support intended actions (Barbalet, 2011). To reach a feeling of rational ease, a person needs to trust the possibility to reach her goals in the first place, as well as feel confident of her own ability to effectuate them, not to get stuck in self-doubt or anxious side tracks (see further Barbalet, 1998: 49). In complex rational deliberation, disruptive emotions such as uncertainty and doubt play a fundamental role and the sense of obviousness or ease can be understood as an ideal endpoint to a process of critical reflection, seeking and stressing potential obstacles as well as balancing consensus and conflict (Bergman Blix, 2022b).

Philosophical research has conceptualized ‘epistemic emotions’ as those involved in knowledge seeking and deliberation. These are vital for evaluating ‘the quality of one’s knowledge, on the extent of what one has learnt, and how much confidence can be placed in what one believes’ (de Sousa, 2009: 140)^3^. In other words, epistemic emotions have ‘epistemic ends’ (Morton, 2010: 386) and influence our convictions and beliefs as well as our inferential strategies or cognitive processes. Curiosity, interest and wonder captivates the mind and sparks new lines of inquiry. Doubt and epistemic anxiety invite reassessments of beliefs and knowledge already acquired, while the feeling of certainty and conviction intermit deliberation and involve saturation (de Sousa, 2009; Terpe, 2016). In court, doubt is an expected and even required emotion (as depicted in ‘without probably doubt’), and studies have shown doubt to be imperative in legal decision-making (Törnqvist and Wettergren, 2023; Minissale and Bergman Blix, forthcoming). Previous theorizing conceptualizes uncertainty as a meta-cognitive emotion that covers both doubt and epistemic anxiety (Arango-Muñoz, 2013), but for our purposes, we need to distinguish between *doubt*, as building on stable alternatives for evaluation (de Sousa, 2009), such as when doubting whether a statement is true or not, and *uncertainty* as lacking evident ways to tackle the problem. This means that uncertainty relates to dejection and confusion and therefore leans more heavily on the collective to be solved. In legal decision-making, uncertainty can arise when legal professionals need to assess legal matters with unclear or imprecise interpretative frames, such as when deciding on intent and credibility (cf. Minissale and Bergman Blix, forthcoming).

So far, our focus has been on subjective experiences, but the way people assess information and evaluate their beliefs are socially learnt and build on social responsiveness and sanctions (de Sousa, 2009). A social basis for cooperation and decision-making in collective settings is trust. Trust is a fundamentally social emotion in the sense that trust both presupposes and generates a bond between persons. Trust is a future-oriented emotion centring around a belief or feeling that another person is reliable, implying that trust comprises risk-taking, some form of dependence and commitment (Barbalet, 2019) that relaxes one’s ‘self-protective strategies’ (Nussbaum, 2016: 94). To trust someone involves a belief in the trusted person’s competence as well as their honesty (Hawley, 2017; Barbalet, 2019) and thus, distrust entails a form of moral criticism. Notably, Hawley (2017) distinguishes between distrust and low expectations and contends that while distrust involve stronger emotional and normative elements, low expectations rather implicate a practical adaptation to material matters. In the collective ritual of deliberations, we find trust and mistrust to be foundational for the epistemic process. Entering the deliberation, generalized trust or mistrust towards fellow judges or lay judges shape the form of reasoning and openness to reveal the epistemic process, while trust or distrust towards specific judges and lay judges can influence the way the epistemic process unfolds. Trust or distrust can also result from the interaction throughout the deliberation^4^. For analytical clarity, we call this latter form *momentary trust* in contrast to the *generalized trust* that serves as a starting point. As a general rule, judges express generalized trust in fellow professional judges, and generalized mistrust or constrained trust in lay judges. Since lay judges do not have legal training, the professional judges generally take a firm hold of the deliberations to ensure that the discussion sticks to relevant issues (Bergman Blix and Wettergren, 2018).

In order to zoom in on the social interactional process of legal decision-making, we employ Collins' (2004) concept of *interactional ritual chains* (IRC). The notion of chains emphasizes the temporal unfolding of decision-making where previous actions and interactions influence expectations for present and future actions, such as entering a panel with feelings of trust or mistrust for the other judges in the panel or feeling high or low emotional energy depending on previous meetings as well as expectations of being in the centre or periphery of the meeting. In social interactionist theory, meaning-making, how we understand situations and form norms and moral boundaries for how to act, are created in meetings with other people, and this link between interpretation and interaction accentuates the fact that meaning-making is dynamic and can change depending on how a situation develops (Blumer, 1969). We tend to endorse information that most people endorse, i.e., we pull towards consensus (Arango-Muñoz, 2013). In legal deliberations, which adhere to strict procedural rules and aim to apply law to facts, the elementary social arrangement for how to deliberate and how to evaluate facts build on situational agreements based on experience and adaption in the moment. How much evidence is needed to prove an assault, does the fact that the witness is a brother to the victim make him more or less credible, how can a disagreement between the judges foster independent collegiality or conflict? These issues cannot be solved from studying legal rules and regulations, they grow out of situational interpretations in interaction rituals. Interaction rituals, if succeeding, generate social solidarity and as depicted in earlier work (Bergman Blix, 2022b), legal deliberation includes an inherent tension between successful rituals, fostering solidarity, and judicial independence, demanding autonomy. Our interest is to analyze how this tension is managed in actual practice by concentrating the analysis on situations when judges need to tackle epistemic uncertainty. Since the feeling of uncertainty lacks clear options for moving towards feeling certain, it opens up to invite the collective and thus illuminates the tension between ritual collaboration and legal independence.

### Methods and materials

This study is part of an international, comparative research project, including four countries, but this article builds solely on Swedish data. In Sweden, we shadowed and interviewed 47 judges and observed 70+ criminal cases (homicide, fraud, domestic abuse and more) in different stages in the legal process (preliminary investigation, lower and/or appellate court). Due to long-standing presence in the field and networks established in earlier research projects, we were also able to secure access to deliberations. We were interested in the seemingly contradictory foundation for legal decision-making as both independent and autonomous yet collective and interactional. This article builds specifically on data from deliberations from both district and appellate courts where the composition of professional and lay judges varies, making interactional aspects, such as different forms of trust and mistrust salient.

Researching subtle emotional processes in the closed and confidential setting of legal deliberation is a challenging quest that demands inventiveness and delicacy. Emotional displays are varied and delicate to interpret, and it is crucial not to assume that people’s experiences match their emotional expressions (Bergman Blix, 2022a). In the legal professional field, emotions are shunned as distortive and biased and emotional displays, particularly in the Swedish setting are very subtle (Bergman Blix and Wettergren, 2018; Flower, 2020). To overcome these methodological obstacles, this study builds on extensive ethnographic fieldwork embracing observations of proceedings and deliberations as well as shadowing and semi-structured interviews with judges in both district and appellate courts. The methodological triad with court observations, shadowing and interviews allows for diverse perspectives on the collaborative/independent efforts of judges’ decision-making process.

To capture the sometimes elusive emotional undercurrents of legal decision-making, we paid attention to different forms of emotion cues, such as facial expressions, tone of voice, body language and metaphors during court and deliberation observations and shadowing. We combined small talk during shadowing with more formal interviews conducted in close relation to the observed deliberations. The interviews are based on a semi-structured template with questions tailored to judges’ professional vocabulary for emotions. They lasted between 40 min and four hours and were all recorded and transcribed verbatim. Depending on the participants’ interest and the length of the case, we conducted one or more interviews with the judges during the course of the proceeding.

The coding generated a multitude of both inductive nodes from the field, such as ‘formal deliberation’ and ‘decisiveness’, and more deductive theoretical nodes, such as ‘emotion management’ and ‘epistemic emotions’. Our initial analysis drew on nodes related to strategies for decision-making, emotion management, deliberation, discrete epistemic emotions (including doubt, certainty, uncertainty, trust, distrust, hunches and gut feelings) and metaphors. Through close readings of these nodes, we teased out central elements of judges’ multifaceted decision-making processes. Situations that involved uncertainty stood out in their shifting of or intensifying the interactional dynamic, and in the next step, we focused on these situations, and how they evolved during deliberations, in relation to both epistemic and interactional ends.

To illustrate the intertwined collective and epistemic process, we present four deliberations that represent the full sample of uncertainty displays in our data from deliberations in Sweden. We selected two cases from each instance to illustrate the difference in generalized trust depending on the constellation of judges (professional judge vs. lay judge majority). The selected cases also represent different types of legal challenges to illustrate the variation in interactional success and failure. The overall intention with focusing on only four cases was to display the complexity and depth of deliberating uncertainty. In our empirical examples, we have changed details about the cases and use pseudonyms to protect anonymity and a five-year age interval to indicate professional experience.

### Mapping legal decision-making as a collective process

Many decisions in court, particularly juridically simple cases, unfold in a routinized way (Hutton, 2006). When cases follow standardized routes with anticipated options, the decision often appears evident. In Sweden, these deliberations include the presiding/reporting judge describing the case, presenting the relevant legal prerequisites, expected interpretations, and often proposing a judgment for the others to (dis)agree on. Here, emotions of trust are less salient for the deliberative interaction. The standardized route along with the presiding judge’s expert status and firm hold of the presentation of the case and possible options, leave little room for deviations (Bergman Blix and Wettergren, 2018: 124–125). Instead, we zoom in on cases where challenges of juridical or evidentiary matters instigate epistemic uncertainty. Expressing uncertainty presents both social risks and benefits. On the one hand, uncertainty exposes vulnerability by revealing ignorance that can lead to professional shame for not knowing or running the risk of being run over by someone who is already certain, on the other hand, uncertainty can be overcome in dialogue with others. By focusing on situations when judges share their uncertainties about legal or factual issues, what we call *uncertainty exchanges*, we will demonstrate, that the way uncertainty exchanges evolve depend on trust or mistrust between participants. We link generalized trust or momentary trust in fellow judges with shared attention and emotional attuning to overcome uncertainty. A successful uncertainty exchange fosters emotional energy and solidarity and facilitates a legal decision-making process ending in deliberate and eventually settled certainty. When uncertainty exchanges are hampered by mistrust, we instead see epistemic anxiety and alienation.

#### Uncertainty exchange in district court

In our first two examples of uncertainty exchange, we visit the district court, where the professional judges Valdemar and Asta enter their respective deliberations with constrained generalized trust in the lay judges. Their expressed uncertainty develops in starkly different ways, either prompting momentary trust or distrust, depending on how they delineate their uncertainty, and whether the feeling resonates with the lay judges.

The first case from a district court, *the mindless attack*, refers to an assault in public ruled by Judge Valdemar (35+) and three lay judges. The evidence for the attack and how it happened is good, but the question of intent becomes complicated since the defendant has an intellectual disability.^5^ Judge Valdemar’s well-defined and articulated uncertainty about how to evaluate intent allows for an open discussion in his interaction with the lay judges in the deliberation:

Judge Valdemar folds his arms over his chest: “The test we're going to do is: did he [the defendant] have control over his action?” Lay judge 1 agrees that is a difficult question. Valdemar unfolds his arms and tells the lay judges about the Samurai case [a famous supreme court ruling about a man assaulting his partner with a Samurai sword during a psychotic episode]. He has an open stance and gestures the story with his hands. He ends by emphasising that it is unlikely that the defendant in the Samurai case did not know what he was doing at all. Valdemar pauses and Lay judge 1 comments on the fact that the Samurai defendant was psychotic while this defendant has an intellectual disability. Valdemar [with interest]: “I understand *exactly* what you mean, and it is a *difficult question*.” He wipes his face with his hand. “Yes, it's not easy, because what he says here, the prosecutor puts the words in his mouth and the defence too, it's *so uncertain*. I mainly want to look at what has *happened*.” Valdemar now directs all his attention towards lay judge 1.

*The mindless attack*, Judge Valdemar, 35+, deliberation

This extract illustrates an open exchange of uncertainty between Judge Valdemar and one of the lay judges. This is not common in our data from the district court, but shows that Judge Valdemar is uncertain about how to make sense and interpret the question about control over one’s action in this particular case. Valdemar has prepared for this question before the trial by reading up on legal analysis about intent, but the issue still needs to be solved in relation to the particular facts of the case that was presented at the trial. In the start of the extract, Valdemar verbally opens up for discussion, but his folded arms signals independence and a reluctance to open up for the lay judges’ input. When he perceives recognition of his own thoughts from lay judge 1, he singles out this particular lay judge as a confidant, unfolding his arms and directing his struggle to find a perspective or frame from which he can interpret the issue of control of one’s action only towards him. As argued above, in contrast to doubt that is directed towards a certain issue or fact, uncertainty lacks stable alternatives and thus demands more unguarded reflection. Still, the uncertainty here relates to a well demarcated issue. Valdemar is not uncertain in a general way and he does not lose his control over the deliberation, he engages in an open uncertainty exchange through an instance of momentary trust in one particular lay judge.

However, we also have a few instances of judges demonstrating more extensive uncertainty. In the *drunken brawl case*, Judge Asta (60+) loses control over the deliberation in a rather ordinary case of assault. Two groups of underaged drunken youth had a fight and a member of one of them is accused of assault. Judge Asta, ruling with three lay judges, opens the deliberation by referring to one of the alleged incidents: ‘This wasn’t easy, but I can say, that I do think that the kick did happen.’ Lay judge 3 replies straight away that he agrees. Judge Asta’s openness to discuss and reflect demonstrates generalized trust towards the lay judges, but her lack of delineation within a legal frame, and their inability or unwillingness to make room for an uncertainty exchange, invites a power struggle between her and them when they do not share her uncertainty:

Judge Asta refers to the event as “a drunken brawl”, but Lay judge 1 opposes, arguing that the victims felt that they were attacked. Asta replies “I’m a bit uncertain myself, that is why I’m open to talk about this”. Lay judge 3 inserts that the victims didn’t fight because they wanted to fight but because they had to defend themselves, and lay judge 2 agrees, adding that the victim probably would have been “smoother” in his defence is he had been sober.

*The drunken brawl*, Judge Asta, 60+, deliberation

As the quote shows, Judge Asta’s uncertainty is not shared by the lay judges. Instead they take advantage of the vulnerability associated with her uncertainty and seize the opportunity to get their interpretation across. While Judge Asta starts to look in her books for relevant case law, the lay judges continue in the same vain and the discussion soon revolves around moral evaluations of the defendant’s perceived behavior without referring to evidence of any kind. When Asta remains uncertain lay judge 2 asks her what she is hesitating about and Asta says a bit vaguely that it is the situation, referring to her initial comment that the case concerns a drunken brawl. Lay judge 2 replies that it was the defendants’ group that started it by yelling, and lay judge 3 adds firmly that ‘the situation is no excuse!’.

In this deliberation, Judge Asta’s risk-taking in trusting the lay judges eventually makes her lose control and power over the deliberation. Lay judge 3 starts by corroborating Asta’s wavering reasoning, but not her uncertainty. Instead, her uncertain stance makes him gain agency for his reasoning and soon the other lay judges agree with his confident stance. It can be noted that lay judge 3 demonstrated confidence in his decision from before the trial, he did not take any notes and sat with his arms folded across his chest the whole trial, but he needed Judge Asta’s expressed uncertainty to gain agency. Her uncertainty made her lose her presiding authority and lay judge 3 could gain emotional energy and self-confidence to go against her (Collins, 1990).

In both *the mindless attack* and *the drunken brawl*, the professional judges entered the deliberation with a constrained generalized trust towards the lay judges as a base for articulating feelings of uncertainty to stimulate further knowledge seeking. As a future oriented emotion (Barbalet, 2019), trust is necessary for any form of knowledge exchange during deliberations. Lack of trust can signal doubt in others’ competence or sincerity (Hawley, 2017), and the Swedish lay judge system invites trust in sincere public participation, while calling for mistrust in competence, since lay judges lack legal education. This ambivalence between trust and mistrust (Hawley, 2017) is indicated in Judge Valdemar’s restrained body language (arms folded) at the start of the deliberation, with a gradually more open body language when he gains trust from the lay judge reflecting his own uncertainty (cf. Nussbaum, 2016: 94). In contrast, Judge Asta’s unrestrained uncertainty forces her into an interaction depending on discretionary trust, and on the way she exposes her vulnerability and loses in authority. The uncertainty exchange between Judge Valdemar and one lay judge was delineated by a legal prerequisite (control of one’s actions) and amounted to a sharing of uncertainty between Judge Valdemar and one of the lay judges, while Judge Asta’s uncertainty around an empirical fact (trying to dissect a ‘drunken brawl’), along with lay judges who felt confident in their certainty when entering the deliberation, grew wider during the interaction. This shows that uncertainty exchanges may build trust and amend uncertainty (mindless attack) while failed uncertainty exchanges can lead to status degradation, growing epistemic anxiety, and distrust (drunken brawl).

#### Shared attention, emotional attuning and emotional energy in appellate court

Turning to the appellate court, the trust conditions for uncertainty exchanges are different. In contrast to the district court, where the professional judge is in minority, they are in majority in the appellate court and share a generalized trust in their fellow professional judges’ competence and sincerity (Hawley, 2017). Professional judges can of course dislike or distrust individual colleagues, but the long route to become a judge in the civil legal tradition paves the way for institutionalized trust in professional adeptness. Our analysis shows that the judges in a panel need to collectively create a *readiness* to decide during deliberations. To overcome uncertainty, the deciding momentum (readiness) builds on shared attention, i.e., joint efforts to dissolve remaining uncertainties, and emotional attuning. Emotional attuning refers to the transient emotional checking in with the people in the deliberation, which is used to validate own interpretations and assessments in relation to the other judges.

Turning to a case of gross violation of women’s integrity,^6^ we show how shared attention and emotional attuning converge into emotional energy and epistemic relief. The case involves Judge Pernilla (35+), reporting judge, senior Judge Bo (50+), chair, Judge Mårten (45+), and two lay judges. In interviews, all judges frame the case as typical for intimate partner violence and state that the case was rather straight forward and ‘easy’. We call this case *the ordinary puzzle*^7^. When we enter the deliberation below, the judges have arrived at the seventh charge in the indictment. They have agreed on the defendant’s guilt on all of the charges discussed so far, which has infused a positive energy into the deliberation (Collins, 2004), building confidence and momentary trust in the group. The seventh charge concerning a minor assault where the defendant allegedly pushed the victim, disrupts their decision flow, and opens up for an uncertainty exchange:

A bit into assessing the seventh charge, Pernilla turns to the defendant’s story about the incident. Teasing out how the different events during the day are bound up, she exclaims “I just don’t get it!”. Judge Bo, the chair of the trial, frowns and says that “Don’t they have two totally different stories?” The third judge, Mårten, starts reading the ruling from the district court, and so does lay judge 1. Lay judge 2 joins the discussion about what has been said about the defendant pushing the victim and when it eventually took place during the day. Lay judge 2 raises another question about the series of events the current day. No one can answer his question and for a while, everyone is silent. Judge Bo then says that they need to account for a situation from earlier during the day to understand how the victim acts in this situation, they need to consider that the defendant blames the victim for getting caught by the police driving drunk, “he is mad at her”. With a firmer voice he says that “It is not *her* being angry with him, it’s *him* being angry with her” and this explains the situation. Both judge Mårten and Pernilla react strongly to this piece of information, they seem to get an epiphany from realizing how this charge relates to the earlier event. Bo concludes by asking if they all agree that the victim is pushed in this charge and that the defendant should be sentenced for a minor assault. No one disagrees.

*The ordinary puzzle,* Judge Pernilla, 35+, Judge Bo, 50+, Judge Mårten, 45+, deliberation

In this excerpt, we can see how hampered sense making relating to contradicting oral evidence feeds into feelings of uncertainty and even frustration, displayed by Judge Pernilla. As all the judges seem to share the uncertainty raised from the incoherence between the testimonies given by the respective parties, Pernilla’s initial outburst invites interest and orients the group’s attention and discussion towards this particular issue (if and when the push takes place).^8^ When judge Bo finds a common-sensical explanation for the reason behind the defendant’s anger, their uncertainty can be resolved: ‘It is not *her* being angry with him, it’s *him* being angry with her’, and all judges immediately agree that the defendant committed the offence. Although many aspects of the actual offence remain unclear, their shared feelings of uncertainty turn into epistemic relief and emotional energy. In contrast to how Judge Asta’s unreciprocated uncertainty in *the drunken brawl case* lead to the deliberation falling apart, the open display of ‘I do not understand’ in this case, invited joint reflection from all the present judges (including the lay judges who were in minority). Their shared uncertainty generated a collective effort to solve the puzzle and when the right piece was found, all judges displayed a readiness to decide. Essentially, this episode demonstrates how uncertainty exchange fosters momentary trust and emotional energy.

In another case in the appellate court, concerning a man who is accused of attempting to murder his wife, the *nonvalid evidence case*, we can see a similar uncertainty exchange leading into a collective build up (shared attention and emotional attuning) prompting a readiness to decide and reach a judgement. In this case, we meet the reporting Judge Ester (45+), the presiding Judge Albert (45+), Judge Felicia (30+), still under training, a court clerk and two lay judges. This case involves several legal issues that makes it unusual and arduous to handle. In addition, the victim, the wife of the defendant, was apparently afraid to talk about the incident and her testimony is restrained in details, while the corroborating evidence is weak. Overall, the deliberation is characterized by hesitation and several successive protracted uncertainty exchanges. Assessing the question of intent, the discussion eventually zooms in on how to evaluate the (lack of) supporting evidence. Judge Ester emphasizes that the weaknesses of supporting evidence is ‘quite unsatisfying’, wordings that echoes throughout the deliberation, pointing towards a lack of lucidness that generates uncertainty:

Turning to the other judges, Judge Ester says: “I would like to discuss this as an open issue”. Judge Albert affirms Ester’s overall assessment of the evidence, but also tones down her worry, assuring that they are not lacking any important information to make a well-founded decision. When saying this, he looks at Ester with a gesture of open hands and finishes the sentence with higher pitch as in a question. Ester repeats: “I am very open for discussion, I have not made up my mind”. The discussion moves back and forth as all judges and lay judges give their input on the case. On several occasions, someone in the panel say that they think there is enough evidence to find the defendant guilty. Coming back to the low evidentiary value of the corroborating evidence, the clerk joins the discussion and argues that “It falls back on the prosecutor” and that “It is not sufficient investigation to come to that conclusion” [that the defendant had the intent to kill his wife]. No one responds immediately and the clerk goes on talking for a while longer. Eventually, judge Felicia says that she is prepared to agree, stating that she now sees the weaknesses of the evidence against the defendant in a different light than before. While there is no verbal agreement to acquit the defendant, after this point everyone makes statements in this direction, emphasizing “beyond reasonable doubt”, and when judge Albert says that they need to release the defendant from detention, no one disagrees.

*The nonvalid evidence,* Judge Ester, 45+, Judge Albert, 45+, Judge Felicia, 30+, deliberation

Judge Ester’s repeated invitation, ‘very open for discussion’, is a common way for judges to signal uncertainty. The phrase demonstrates a leap of trust towards an uncertainty exchange, involving emotional attuning with the other judges in the deliberation, as Ester wants to know their stance on the case to move her own thinking forward. Being the reporting judge, Ester has the greatest responsibility for the preparatory work paving for a focused and successful discussion. Her exposed vulnerability by inviting an uncertainty exchange at this point of the trial is met with affirmation and cautiousness in the following discussion. Judge Albert tries to build a collective readiness to decide [assuring that they have all the information they need] while being careful to not shut down Ester’s uncertainty quest [open hands combined with higher pitch as in a question]. In this exchange, emotional attuning is manifested through a clear interest in other judges’ point of view but also a seemingly strong search for consensus.

An important difference compared with the *ordinary puzzle case* analysed above, is the lack of epistemic relief or emotional energy in reaching this decision. Instead, the atmosphere is saturated by anxiety and solemnity burdened with responsibility. At the end of the deliberation both Albert and Felicia articulate their belief that the defendant actually intended to kill the plaintiff but assure that their *beliefs* are not enough to hold him responsible. The decision to acquit the defendant ends in joint concern for the victim:

But we can't use that to convict him. I think it's too weak. But I think it's difficult because I have my thoughts about how it's really like and I think she's in great danger and it doesn't feel good.

*The nonvalid evidence*, Judge Felicia, 30+, deliberation

While it is common in our data for judges to say that it is easy to acquit, emphasizing the rule of law and stressing the risk of convicting an innocent person, in this particular case, dismissing the case also entails a strong worry for the plaintiff’s safety. The severity of the crime and the personal beliefs of *what really happened* take away some of the emotional energy and confidence that mutual decisions commonly entail. While Collins (2004: 49) stresses moral righteousness as an outcome of successful rituals, the emphasis on a separation between moral versus legal evaluations in the (Swedish) civil legal system seem to hamper emotional energy and solidarity, at least momentarily. The judges’ moral investment lie in following a correct legal procedure, but their concern for the plaintiff’s welfare blocks the emotional energy that their joint ‘correctness’ should entail.

#### Reaching autonomous certainty, solidarity or alienation

Our examples so far have illustrated uncertainty exchanges as profound to the judgment of the case. As we have seen, uncertainty exchanges, in many instances, move the deliberation closer to feeling certain. However, as we will turn to next, in legal decision-making, certainty is an agile and versatile emotion. Even when legal professionals feel certain already when entering the deliberation, this feeling needs to be thoroughly scrutinized due to the high demands on objectivity and impartiality. In fact, in our overall material, most judges are already *leaning towards* a decision when entering the deliberation, demonstrating what we here call *agile certainty*. Agile certainty is highly valued among the judges in our study and judges emphasize that during court proceedings their opinions of a case ‘can turn a bit all the time’ as ‘you get different pieces of the puzzle’ (Lydia, 35+, appellate court). In the deliberations, discussions that promote ‘twists and turns’ are seen as stimulating, and disagreement, as well as a reliance of discussions to further, or even break, one’s chain of thoughts ‘foreshadows for quality in judging’ (Ruben, 55+, appellate court). Our analysis shows that the possibility for uncertainty exchanges during the deliberation is seen as a crucial stepping stone to secure a feeling of having reached a thoroughly reflected decision, a *deliberate certainty*. While deliberate certainty indicates readiness to decide, it does not necessarily entail the conviction and confidence of *settled certainty*. For the feeling of certainty to settle, the judges at times need temporal distance from the uncertainties and nuances that have aroused during the deliberation.

Similar to the uncertainty exchanges, these different levels of certainty are formed in collaboration with the other judges as a way to ensure independent decision-making. Illustrating the dependence of the collective for moving through different levels of certainty, we return to judge Bo in the *ordinary puzzle case*. Valuing the input from his fellow judges, he says that the other judges made him more confident about his interpretation of the evidence in a charge where the defendant was accused of throwing a mobile phone on his partner:

I decide…I would say I make up my mind when I decide in the deliberations. Because I want to hear everyone else's possible views. For example, now when the lay judge said “It must have been a hard throw”. Well, that was good, it's an argument for *my* standpoint and before that I hadn't finished those thoughts.

*The ordinary puzzle*, Judge Bo, 45+, post-hearing interview

This quote shows how Judge Bo came to the deliberation feeling agile certainty (‘I want to hear everyone else’s possible views’). His agile certainty moves into a deliberate certainty as confidence about his standpoint is gained through a collective confirmation (‘when the lay judge said “It must have been a hard throw”’). Accounting for his independent decision-making process, Judge Bo uses the personal pronoun (‘decided’), however the collective has a central role as the panel assisted him in ‘finishing [his] thoughts’. As we can see, the input from the other judges can bolster the judge’s sense of autonomy, resulting in *autonomous consensus* where both the individual and the collective are accentuated. Judge Bo’s deliberate certainty is saturated with confidence and ease. For this seasoned judge, balancing independence and collaboration seems effortless. The more junior Judge Pernilla, the reporting judge in the same case, is more cautious in articulating this balance. Her collaboration leans on gratitude towards the senior judges in the panel since she, as a junior judge, ‘learns things all the time’. Emphasizing that judges Bo and Mårten are ‘diplomatic’, she assures that:

Sure, you can be dissenting if you want to, if you have a very [lowers her voice] strong … opinion of something. It may sound wrong to say that … that you shouldn't be [breathes in heavily] [dissenting]. If you want to rule in a certain way, you should DO SO. But there is a point to… [hesitates, resigned] that one can reason with both of them [Bo and Mårten]. They are open to reasoning. An open-minded atmosphere [raises her voice] and you can also sometimes be the devil's advocate and turn things around [lowers her voice]. But also, very pragmatic.

*The ordinary puzzle*, Judge Pernilla, 35+, post-hearing interview

In this quote, we can see how Judge Pernilla balances the value of judges’ autonomy and independence with more collegial and pragmatic aspects of the legal decision-making process. Pernilla expresses a threshold for having a dissenting opinion, yet her breathing and lowered pitch indicates that this pull toward consensus (Arango-Muñoz, 2013) is a challenging opinion as it counters the ideals of legal independence and autonomy. While pointing towards individual space for decision-making (‘an open minded atmosphere’) and room for opposing assessments (‘playing the devil’s advocate’), judge Pernilla also underscores trust and solidarity with her fellow judges as they are both ‘open to reasoning’. Trusting that her fellow judges care for her independent process, increases solidarity with both with the panel of judges and their decision. Taken together, the quotes by Judge Pernilla and Bo are elucidative examples of how independence, as embedded in the social interaction of deliberations (Bergman Blix, 2022b), can produce solidarity (Collins, 2004).

However, not all deliberations end in solidarity and bolstered autonomy. For comparison, we return to Judge Asta in the district court and the *drunken brawl case*. Asta was uncertain about how to interpret ‘the situation’, but eventually resigned and agreed on a guilty verdict. As a professional judge, she is *not* uncertain about sentencing in these simple cases and in most deliberations, lay judges leave these assessments for the professional judge to decide. In this case, the most opinionated lay judge 3, nonconformally wants to maximize the possible fine. Asta tries to reason with him, arguing that the predefined sanction levels should be applied. When he refuses, she turns her back to him and tries to reason with the other lay judges: ‘But what do you *think*?!’ When that does not work either, she gives up but expresses her frustration: ‘*Screw it*, we’ll give him the higher amount then. I’ll feel bad about it, but we have to pass the judgment.’ Asta tries to regain control, but fails and decides to go along with the unusually large fine. Since the lay judges’ arguing based on personal opinions worked at first, the discussion gets stuck in this moral frame devoid of legal relevance. For Judge Asta, the deliberation becomes a struggle and exposing her uncertainty leads to diminishing her autonomy. In this situation, Asta started out trusting her fellow judges, but ended in stark distrust and conflict. In an interview a week later, judge Asta takes the blame for the disagreement herself:

[laughing] I can almost feel ashamed, because it was ridiculous in a way I think … and it turned into an unfortunate discussion, because I tried [to convince the lay judges] for quite some time, and I should have stopped it earlier, but now I didn’t. […] Sometimes stupid wins. That’s how it is, and there was nothing I could do about it.

*The drunken brawl*, Judge Asta, 60+, post-hearing interview

The resignation and lack of agency in this quote is telling of a collective process ending in non-solidarity and alienation. The failing uncertainty exchange impedes the possibility to reach a deliberate certainty and the decision becomes associated with professional shame and individual failure. The interaction between Judge Asta and the lay judges culminate in hollow consensus where pragmatic considerations conquer independence and autonomy.

Going back to Judge Ester and the *nonvalid evidence case*, the collective process during the deliberation steers her epistemic process in another way. In an interview after the court proceeding, judge Ester depicts her struggling with whether the defendant, the man who was accused of attempting to murder his wife, should be acquitted or not. Describing how she felt that the weaknesses of the evidence got more and more worked in’ during the deliberation so that, in the end, she felt certain (deliberate certainty) that they should acquit the defendant. In an interview the week after, her anxiety about the decision (as depicted in a previous section) is gone. Trusting the process, Ester reckons that:

But IN the deliberation, I was probably still a little unsure about [*the defendant*] in particular, I *was,* that was what I was *struggling* with. Because I had felt before that, or it was my thought that, that it was probably still *enough* [evidence to find him guilty]. Erm, but then I think we had a good discussion there as well, and everyone also brought up the insecurities everyone HAD about this and then I think it's… it WAS exactly like, like it turned out to be [acquitting him]. […] And, it makes me feel confident in that I cannot do this any other way, any other way would turn out *completely wrong*, and that makes it so much *easier after all*.

*The nonvalid evidence*, Judge Ester, 45+, post-hearing interview

One week after the deliberation, Ester still remembers her initial leaning towards (agile certainty) finding the defendant guilty, and her own uncertainty struggle when they scrutinized the evidence during the deliberation (‘a good discussion there as well, and everyone also brought up the insecurities’). At the same time, the judges’ collective anxiety and moral worries that characterised the ending of the deliberation (as seen in the quote by judge Felicia on page seven) are gone and replaced with settled certainty and assurance (‘it makes me feel confident’, ‘any other way would turn out *completely wrong*, and that makes it so much *easier after all*’). When everyone has shared and discussed their own uncertainties, they feel that they have sorted it out from multiple perspectives, the collective effort makes the decision seem evident. At this point, her decision is imbued with a naturalized ease and confidence that closes further inquiry. The high amount of issues that generated uncertainty along with moral worries for the victim that hindered collective emotional energy and epistemic relief at the end of the deliberation, have now receded into the background, providing room for professional pride in doing a good job (Ester, 45+): ‘You feel confident with how to DO in an objective, legal way’. In retrospect, the collective deliberative process produces trust in her decision and reproduces solidarity with her fellow colleagues; together they can form legally correct (‘in an objective, legal way’) autonomous decisions.

## Discussion

The ultimate aim of legal deliberations is to take decisions, and previous research has shown resoluteness and decisiveness to be valuable assets for individual judges, while dwelling or regret over made decisions are considered inapt (Törnqvist, 2017; Bergman Blix and Wettergren, 2018). As we have argued in earlier work (Bergman Blix, 2022b), the legal decision-making process is grounded in micro-decisions and the legal procedure in itself provides a structure where judges’ decisions are mapped out in different sections. When judges have brought one question to an end by reaching certainty about an issue, they are able move forward in the decision-making process, usually by going into uncertainty or doubt regarding another question. Looping in and out of uncertainty/doubt and certainty becomes a prominent feature of the legal decision-making process (Törnqvist and Wettergren, 2023).

In this article we show that legal decision-making needs to balance uncertainty and certainty through ‘uncertainty exchanges’. If the judges get stuck in uncertainty, they lack the vigor to decide, but if they solely feel certainty, they accept any proposal without scrutiny (cf. de Sousa, 2009). This emotional balance applies for legal challenges^9^, as in the evaluation of intent in the *mindless attack case*, as well as challenges due to evidential weaknesses, as in the *ordinary puzzle case*. When judges rule in panels, building momentum to decide becomes a collective enterprise. By adapting Collins' (2004) interaction rituals, we link shared attention, emotional energy and solidarity to this balancing act, and demonstrate the significance of trust to understand ritual success or failure. In sum, epistemic emotions underpin legal decisions, and independent decision-making demands a collective effort.

The first two parts of our analysis focus on uncertainty exchanges in deliberations in lower and appellate court settings. While doubt is regarded as a crucial resource in legal decision-making and highly valued by the judges in our study, uncertainty involves vulnerability and jeopardize core legal values as autonomy and independence. To understand how uncertainty exchanges unfold in legal deliberation trust becomes key. Previous theorizing has stressed self-trust as important for rational action (Barbalet, 2011; James, 1879), a trust in one’s ability to perform and find a solution. We find that in these collaborative settings, social trust is also important for the drive to stay open-minded, and to negotiate a collective, autonomous decision. Trust frames the uncertainty exchanges both as an entry point, referring to a *generalized trust or mistrust* for professional and lay judges respectively, and as an interactional achievement, generating *momentary trust* or *distrust*.

Uncertainty exchanges are potent vehicles for judges to move from uncertainty into certainty by exploring difficult legal issues collectively. Through shared attention and emotional attuning, successful uncertainty exchanges render emotional energy and epistemic relief, emotional undercurrents needed to create a readiness to decide and reach a judgement. As our data shows, uncertainty exchanges sometimes fail, turning into power struggles, diminished autonomy and growing distrust. In these cases, emotional energy and confidence is replaced with professional shame, alienation, and forfeited pragmatism. In other cases, uncertainty exchanges succeed in that they lead to deliberate certainty, where legal matters have been collectively solved and agreed on, yet the emotional energy is low since the outcome of the legal decision is perceived as morally ambiguous.

Our last analytical part explores the role of certainty in legal decision-making. Earlier theorizing argues that certainty ‘freezes inquiry’ (de Sousa, 2009: 146), it is a feeling one wants to hold on to. As we show, in legal decision-making, certainty follows a more complex route, both in the need to delay and scrutinize one’s certainty, and in demands to balance independent certainty with collaborative consensus. Certainty should be agile during deliberations, and deliberate in forming decisions. The latter results in a potential temporal gap between deliberate certainty as a valid inference to take action (decide), and settled certainty, saturated with confidence and ease, when imminent uncertainties and moral consequences have subsided.

By analysing epistemic emotions, primarily certainty and uncertainty, at play in a collective setting, we show that these emotions coalesce with and depend on social emotions of trust and solidarity. Both generalized and momentary trust in fellow judges is shown to bolster autonomy. The emotional energy from the collective deliberative process produces trust in own (independent) decisions and (re)produce solidarity with fellow colleagues.

In relation to our findings, we would like to propose three endeavours for future research. First, interactions in the legal decision process form in and through power and status relations. A closer scrutiny of how social positions influence the interactional frames of legal deliberation and link with rational emotions of ease and confidence may provide important insights to how trust/distrust and uncertainty/certainty develop. In deliberations, uncertainty is seen as productive means to substantiate knowledge, but it is also entwined with vulnerability. Since trust infuse social power between the dis/trusting and dis/trusted persons (Hawley, 2017), an analysis of status-trust can further our understanding of the dynamic of shared attention, emotional attuning and emotional energy in uncertainty exchanges. Second, our findings suggest that the interface between legal and moral frames can instigate, balance and complete epistemic processes by consorting epistemic and collective emotions. Lastly, and partly overlapping with the previous suggestion for future research, we would like to push for studies to explore how and when morality enters the legal decision-making process. While common sense in many instances provide a safe guard to keep legal decision-making relevant to reality, our data also suggests that it is often in relation to common sense moral evaluations enter. These last two questions emphasize the importance of international comparisons between different legal systems. Since morality and common sense are built into the legal process in different ways in different legal traditions, comparative studies are needed to study how and in relation to which questions moral evaluations and common sense enter into deliberations.

## Data Availability

The datasets presented in this article are not readily available to protect anonymity, and due to confidentiality of legal deliberations. Requests to access the datasets should be directed to Stina Bergman Blix, stina.bergmanblix@uu.se.

## Footnotes

^1^If there is a two against two vote in the district court, the decision most lenient towards the defendant will be the decision in force.

^2^It is important to note that the perception of rationality does not assume that the action is correct or reasonable in any absolute sense.

^3^Since emotions are linked to action readiness, all emotions can have epistemic qualities, for example anger makes us more prone to attribute blame and take fast decisions, but is seldom defined as an epistemic emotion. For epistemic emotions, the action readiness is primarily cognitive as when evaluating information or settling on a decision. They guide and motivate without articulate expressive features or stirring attention in their own right.

^4^While mistrust and distrust largely convey the same meaning in vernacular language, in this article we distinguish between them for analytical purposes. Mistrust stands for a general suspicion and skepticism. Distrust, on the other hand, accounts for a settled stance, usually based on experience or concrete information about the other person.

^5^From 1965, the accountability requirement is no longer part of the Swedish Penal Code. A defendant suffering from severe mental disorder or disability is prosecuted on the same grounds as a sane defendant and potential adjustments are made at the sentencing stage. This differs from most countries where accountability is seen as a prerequisite for criminal liability. The suspension of the accountability requirement has complicated the assessment of intent in these cases, in particular with regard to the assessment of the defendant’s subjective awareness of the situation and their actions. In general, the threshold for awareness is set very low and includes actions that are not purely reflexive.

^6^Gross violation of women’s integrity is a Swedish criminal construct aimed at repeated intimate partner violence. In this particular case, the indictment includes several charges, mainly assault and minor assault.

^7^Despite instantiating an ordinary puzzle, the case also contained typical features of ‘word-against-word’ with a general lack of eye-witnesses to the incidents, which lead to uncertainty regarding several issues during the deliberation.

^8^Note that this question is very similar to the issue in the drunken brawl case.

^9^The emotional dispositions involved in the epistemic process of interpreting legal texts can be seen as more complex since it evolves during a longer temporal perspective, including the training process in law school and in judge training.
